# Supplementary material for: Multimodal analysis of cell-free DNA identifies epigenetic biomarkers for amyotrophic lateral sclerosis diagnosis and progression
Source: J Clin Invest. 2026 Jun 1;136(11):e191508. doi: 10.1172/JCI191508 (PMC13221225; doi:10.1172/JCI191508)
Supplement: Supplemental data [file jci-136-191508-s192.pdf]

Supplemental Table 1. Tissue deconvolution from blood-derived cfDNA

Participant	Neural (%)	Muscle (%)	Blood-immune (%)	Connective (%)	Epithelium (%)	Sex-related (%)	Other (%)
Control_1	3.17	4.76	39.37	5.30	16.42	8.90	22.07
Control_2	4.45	6.93	40.67	4.19	15.63	8.54	19.59
Control_3	5.26	6.89	36.00	5.77	16.69	8.58	20.82
Control_4	3.51	5.98	46.28	6.32	9.45	7.98	20.48
Control_5	3.35	4.11	50.24	4.26	16.59	8.66	12.80
Control_6	4.78	3.96	40.74	3.51	15.30	11.44	20.26
Control_7	3.40	3.88	46.55	7.31	13.89	7.49	17.47
Control_8	2.68	5.07	41.05	5.84	16.77	8.56	20.04
Control_9	3.13	4.43	44.46	7.46	12.68	7.52	20.33
Control_10	2.71	5.02	45.53	6.96	14.38	9.25	16.15
Control_11	4.21	5.57	37.41	6.52	19.44	7.14	19.70
Control_12	7.06	4.20	49.17	6.42	10.33	7.23	15.60
Control_13	3.96	2.82	46.65	6.75	13.52	9.56	16.74
Control_14	3.29	3.60	44.72	8.06	14.61	8.42	17.31
Control_15	2.24	4.65	39.92	4.38	16.24	9.54	23.03
Control_16	3.08	3.97	55.15	4.16	11.62	7.71	14.31
Control_17	5.23	3.78	37.08	3.74	23.23	7.64	19.29
Control_18	4.42	6.55	38.97	3.59	13.60	9.23	23.64
Control_19	6.24	6.51	46.61	5.73	11.65	8.45	14.80
Control_20	5.00	4.34	55.71	3.25	9.43	5.76	16.51
Control_51	5.41	5.15	36.31	6.98	13.11	8.94	24.10
sALS_21	3.61	5.09	39.73	6.30	16.27	9.85	19.15
sALS_22	5.14	3.42	35.70	9.12	16.86	10.50	19.27
sALS_23	4.92	4.19	40.89	4.91	15.88	8.89	20.32
sALS_24	4.82	4.16	39.86	8.08	13.57	9.31	20.20
sALS_25	4.73	5.08	39.37	4.83	15.30	10.29	20.40
sALS_26	3.98	1.91	71.11	3.02	4.54	6.78	8.67
sALS_27	4.96	4.87	35.82	9.31	10.97	7.56	26.50
sALS_28	6.81	4.16	40.76	6.10	15.59	9.16	17.41
sALS_29	4.15	4.14	40.23	5.29	14.54	11.91	19.74
sALS_30	5.76	6.12	38.03	4.64	13.85	10.38	21.22
sALS_31	3.06	2.69	49.39	9.19	13.02	6.59	16.06
sALS_33	3.65	3.15	51.20	8.81	9.66	7.30	16.24
sALS_34	5.40	4.30	38.85	7.30	13.74	7.10	23.31
sALS_35	1.32	5.21	48.16	6.02	9.37	10.13	19.79
sALS_36	1.38	5.14	42.83	6.69	15.66	9.47	18.84
sALS_37	4.71	3.88	51.33	5.30	11.10	9.67	14.01
sALS_38	3.88	4.26	41.59	6.17	16.06	10.35	17.69
sALS_39	3.76	4.35	40.54	6.38	15.10	9.51	20.36
sALS_40	2.29	4.18	42.77	8.35	14.74	7.91	19.77
C9-Carrier_41	5.74	4.29	36.69	8.15	16.03	10.09	19.01

C9-Carrier_42	9.65	4.93	41.96	5.27	12.38	8.61	17.21
C9-Carrier_43	1.35	4.57	32.31	5.60	20.94	9.81	25.43
C9-Carrier_44	5.35	4.57	34.12	7.72	13.45	12.90	21.89
C9-Carrier_45	3.68	5.71	44.16	4.86	11.30	10.78	19.51
C9-Carrier_46	4.61	5.79	36.81	6.06	12.97	10.43	23.33
C9-Carrier_47	5.71	3.81	42.94	4.21	15.61	8.83	18.89
C9-Carrier_48	5.21	4.85	37.99	5.50	15.07	11.20	20.18
C9-Carrier_49	0.90	4.65	43.37	6.44	12.93	11.18	20.53
C9-Carrier_50	2.03	5.10	47.23	5.14	15.32	7.35	17.83
C9-ALS_66	3.83	4.94	29.38	5.08	14.96	8.92	32.90
C9-ALS_67	3.62	5.23	49.52	5.71	11.56	7.55	16.81
C9-ALS_68	4.57	4.91	40.48	5.49	12.49	10.34	21.73
C9-ALS_69	6.31	5.77	35.84	6.00	16.22	9.75	20.12
C9-ALS_70	4.26	6.31	37.79	5.69	17.04	9.81	19.10
C9-ALS_71	5.57	3.93	45.56	6.32	14.03	10.26	14.32
C9-ALS_72	5.23	6.13	42.23	6.50	12.79	8.89	18.23
C9-ALS_73	4.51	5.49	43.90	6.91	13.18	9.46	16.54
C9-ALS_74	5.14	5.24	37.86	5.43	17.79	9.69	18.86
C9-ALS_75	4.29	6.09	35.20	6.32	15.59	8.05	24.45

cfDNA = cell-free DNA

Supplemental Table 2. Inter-individual biomarker variability of the top 100 differentially methylated CpG sites

Top N	All	Control	sALS	All	Control	C9-ALS	All	Control	C9-Carrier
50	0.630	0.619	0.621	0.250	0.117	0.160	0.592	0.488	0.750
100	0.591	0.572	0.588	0.242	0.127	0.184	0.559	0.437	0.771
200	0.552	0.530	0.555	0.273	0.177	0.231	0.531	0.439	0.656
500	0.482	0.468	0.468	0.307	0.237	0.279	0.493	0.421	0.579
1000	0.457	0.449	0.433	0.331	0.271	0.319	0.469	0.405	0.538
2000	0.410	0.405	0.383	0.347	0.298	0.337	0.431	0.367	0.502

sALS = sporadic ALS patients, C9-ALS = patients with a repeat expansion in the C9orf72 gene, C9-Carrier = asymptomatic carrier of a repeat expansion in the C9orf72 gene

Supplemental Table 3. Tested ALS mutations and risk factors
ALS2, ANG, ARHGEF28, ATXN2, BSCL2, C9orf72, CCNF, CHCHD10, CHMP2B, DCTN1, ERBB4, FIG4, FUS, GBE1, GLE1, GRN, HNRNPA1, HNRNPA2B1, HSPB1, HSPB8, MAPT, MATR3, MME, NEFH, NEK1, OPTN, PFN1, PRPH, SETX, SIGMAR1, SOD1, SPG11, SPG20, SQSTM1, TAF15, TARDBP, TBK1, TUBA4A, UBQLN2, VAPB, VCP, VEGFA, VPS54

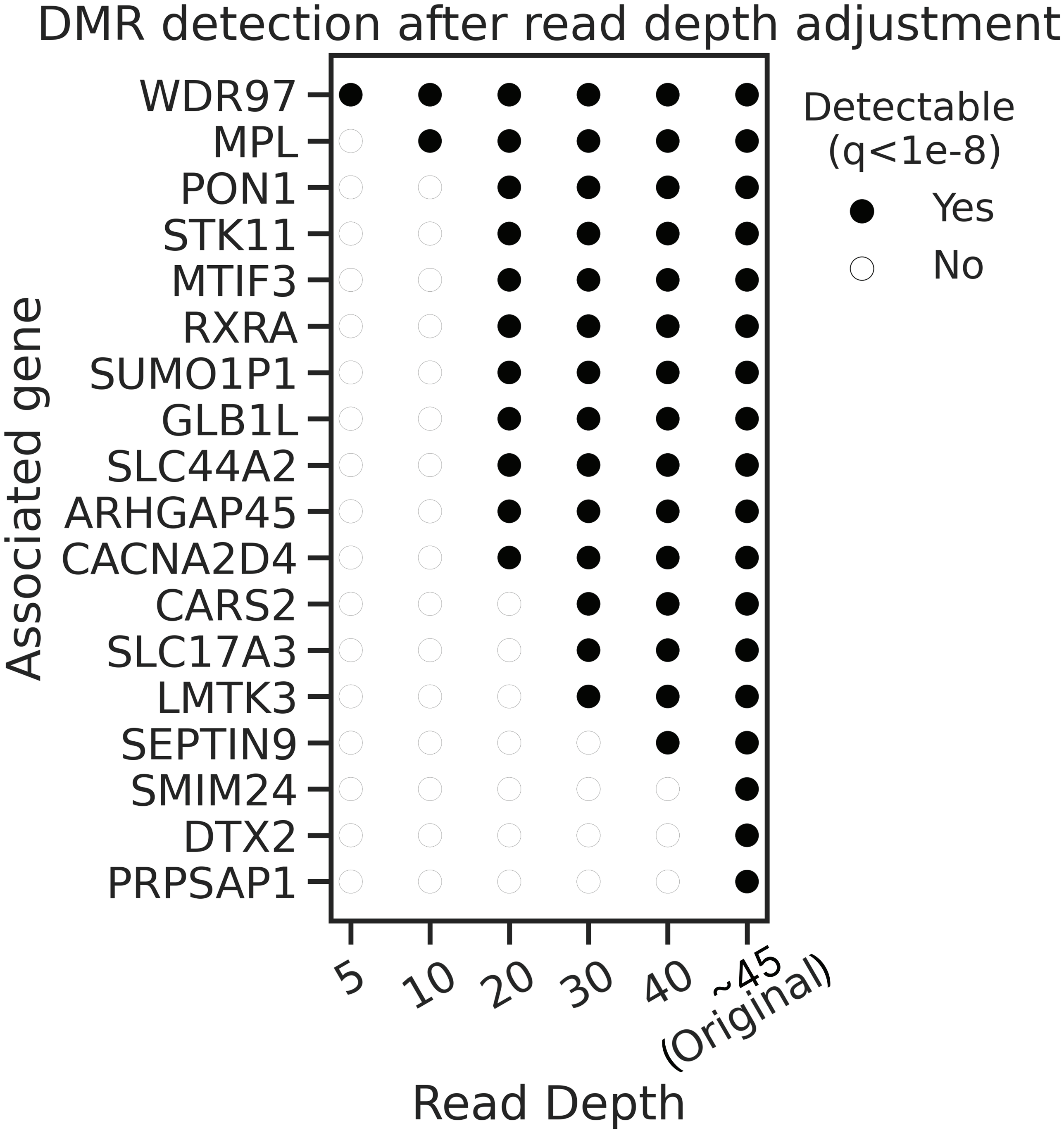

Figure S1. Extent of read depth required for successful detection of differentially methylated regions in epigenetic sequencing analysis of cell-free DNA

Detection of differentially methylated regions (DMRs) at q < 1e-8 across varying read depths (X-axis) for different specific genomic regions (Y-axis). The Y-axis represents DMR-associated genes identified from our original analysis.

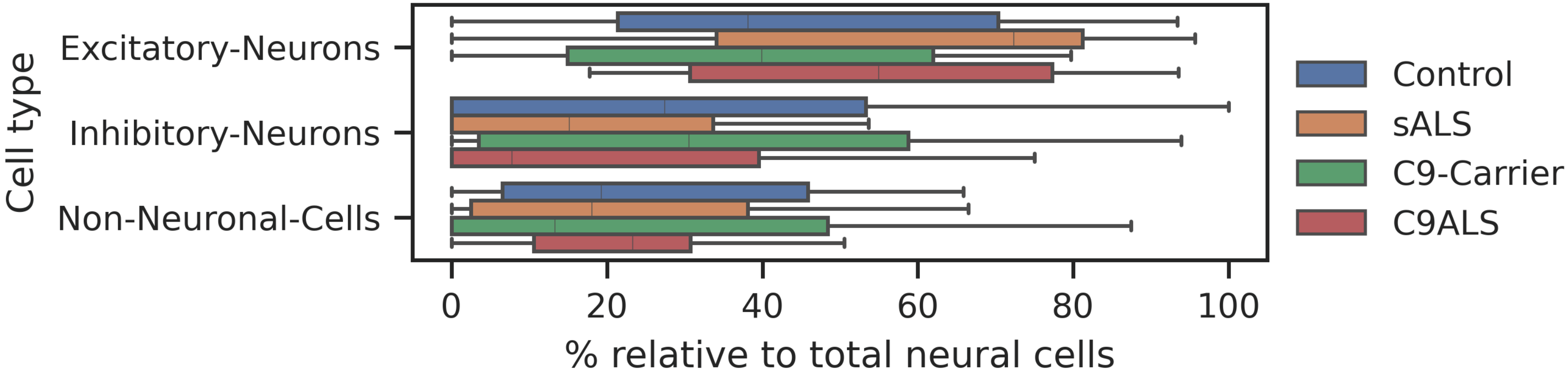

Figure S2. Determination of specific cell type contribution to the neural component of cell-free DNA

UXM deconvolution analysis using an extended reference atlas of 188 brain cell subtypes. Boxplots show that three brain cell types emerge as the major contributors to the neural cell-free DNA signal, with the relative contribution to total neural cells of each major contributing cell type indicated.

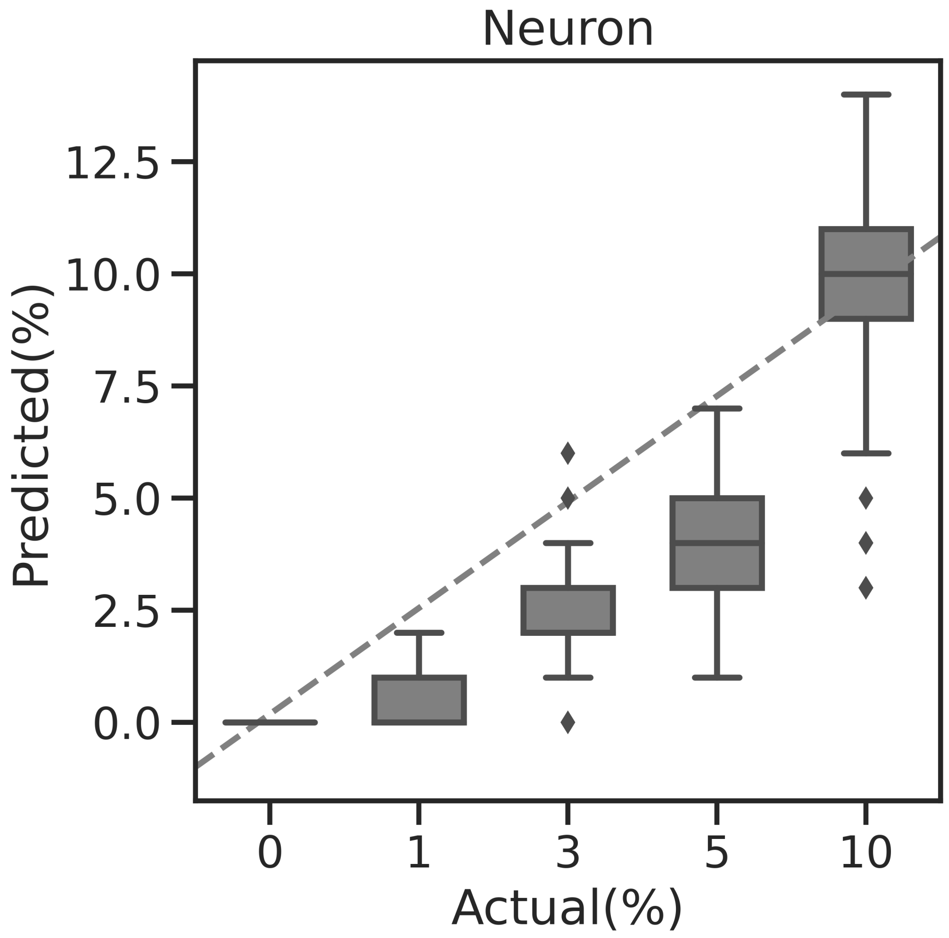

Figure S3. Validation of UXM deconvolution algorithm for determination of tissue type representation for cell-free DNA sample analysis
UXM deconvolution analysis of 50 mixtures of five different neuron samples, where the relative concentration of neuron input ranged from 0 to 10%. Here we see the UXM deconvolution algorithm result shown as ‘Predicted %’ on the Y axis plotted against the ‘Actual %’ concentration of neuron input to the various mixtures. The UXM algorithm was applied to all cell-free DNA samples used in this study to determine their tissue-type composition, which is shown in Figure 2C.
